# Proportionality, Evidence and the COVID-19-Jurisprudence in Germany

**DOI:** 10.1007/s41125-022-00087-7

**Published:** 2022-11-19

**Authors:** Pascal Berger

**Affiliations:** 1 Department for Science Studies, Forum Internationale Wissenschaft, University of Bonn, Heussallee 18-24, 53113 Bonn, Germany; 2 Chair for Technology and Diversity, Institute of Sociology, RWTH Aachen University, Eilfschornsteinstraße 7, 52062 Aachen, Germany

**Keywords:** Science, Proportionality, Law, Politics, Global health

## Abstract

What is proportionate? The measures taken worldwide to contain SARS-CoV-2 deeply curtailed the fundamental rights of many citizens. The courts have upheld this course of containment: the protection of life and health takes precedence over individual liberties, even in the case of doubt about scientific evidence for the effectiveness of far-reaching measures. This finding is astonishing and does not automatically follow from the International Health Regulations, according to which interventions in fundamental rights have to be justified by facts. The principle of proportionality is the logical place where facts and normativity meet. Since science has polarized during the pandemic, the court selection and interpretation of scientific expertise is itself a politicized value judgment. Courts, I conclude, base their selection and interpretation on publicly available and legitimate knowledge. I develop this hypothesis on the basis of COVID-19-jurisprudence in Germany.

## Introduction

Countries around the world reacted to the spread of SARS-CoV-2 with so-called non-pharmaceutical interventions (= NPIs).^1^ The intensity of the restrictions on many freedoms stands out. As late as January and February 2020, international health law experts commented on the quarantine measures taken by China as a historical precedent that would be hard to transfer to Western democracies because of the deep infringements on civil liberties (Raposo 2020). Even during a global health emergency, the law of international health regulations applies, according to which all measures must take into account the principle of proportionality (Habibi et al. 2020).

After the Italian government imposed a mass quarantine on northern Italian cities on February 23, 2020, the German public discussed possible measures. Since democratic politics has to justify its actions on the basis of proportionality, the lockdown^2^ of entire cities seemed to be only a theoretical possibility (Bender 2020). What is considered proportionate quickly shifted. Still considered an inadequate response to the spread of SARS-CoV-2 by the federal government on March 11, external borders and schools were closed nationwide on March 16. By March 23, nationwide closures and contact restrictions were in place, unless previously decided at the state level. The reason for the measures was to avert dangers to the life and health of the population under the impression of exponentially rising infection numbers (Rixen 2020: 1101). In terms of depth and duration, the Federal Republic of Germany experienced the most extensive restrictions on fundamental rights in history.

Judicial proportionality tests are the place where facts and normativity meet in order to subject the political process to scrutiny. Despite their criticism and depth of intervention, the measures adopted since March 2020 have essentially withstood judicial proportionality review. Similar observations can be found worldwide. There have been isolated cases that have bucked the trend, such as the revocation of vaccination status as a means of access to the retail sector. However, none of the most extensive lockdown measures have been overturned by a court. At the same time, successes in court have often been justified less by considerations of proportionality than by formal principles such as the principle of equal treatment. This finding gives reason to analyze the use of scientific knowledge in German courts.

In the following, I will argue that the proportionality test is an arena of negotiation of global health between science, politics and law. In a first step of my argument, I place the elements of this negotiation arena in their general interdependence context, before moving on to the analysis of selected court decisions in Germany in a second step of the argument.

### Proportionality and Evidence in Global Health Governance

Following Max Weber, law, science and politics can be understood as different value spheres with their own rationality, which stand in relationships of mutual restriction and dependence (Terpe 2020). While public health has developed standards of evidence to assess the quality of knowledge, the access of law and politics to scientific knowledge is different. Politics and law have to comply with criteria of public legitimacy and acceptance (for politics see Burstein 2003; for law see van Gestel and Porter 2016: 176–177, Popelier et al. 2021: 2–3). While politics is geared toward winning majorities, jurisprudence is expected to show restraint before democratically legitimized politics.

The restriction of fundamental rights is a constitutionally legitimate means of democratic states. No freedom is absolute (Meßerschmidt 2020: 284–285). Even before the Corona pandemic, the German Infection Protection Act permitted interference with the "fundamental rights of […] freedom of assembly" and "inviolability of the home" (Bundestag 2000: 1056). Restrictions on fundamental rights are imposed to protect other fundamental rights, such as the right to life. In other words, the protective interest (positive obligations by the State) can include protection of the freedoms of others (which may include the right to life and other rights). However, state institutions may not restrict fundamental rights arbitrarily, but are subject to a proportionality test. The proportionality test comprises three levels: (1) adequacy, (2) necessity and (3) the weighing of goods (Meßerschmidt 2020: 287). That means, firstly (1), a measure exhibits a minimum degree of effectiveness in achieving the goal; secondly (2), a measure must not only be effective, but also the comparatively mildest means of achieving it, assuming the same effectiveness; thirdly (3), a means may be effective and the comparatively mildest way of achieving the goal. Then, however, a balancing of interests must still take place between the goal pursued or the good to be protected on the one hand and the interest or good to be restricted on the other (Meßerschmidt 2020: 287).

Global health governance is currently one of the most important application areas of the proportionality principle. The proportionality test is part of the International Health Regulations (= IHR), a treaty-based framework of the World Health Organization (= WHO) for regulating global health protection (Gostin and Katz 2016: 264). The International Health Regulations have their roots in international conventions of 1851 for the control of communicable diseases such as cholera, plague and yellow fever and were last revised in 2005 in response to the SARS-1 outbreak in 2002. The framework aims to balance health, trade and human rights: Member States must provide sufficient scientific evidence of effectiveness before taking certain measures (Gostin and Katz 2016: 266–267). In-line with the principle of proportionality, restrictions should not be more restrictive than necessary. In this sense, at the beginning of the 2020 pandemic, legal scholars warned that measures “must reflect the international law principles of necessity, legitimacy, and proportionality that govern limitations to and derogations from rights and freedoms.” (Habibi et al. 2020: 664).

So where does science come into play? This starts at the institutional level. The International Health Regulations require WHO member states to report and monitor diseases (Gostin and Katz 2016). In the case of emerging diseases, the WHO has an international surveillance and rapid warning function (Robert Koch Institute 2019: 35). National and international health organizations together form a network of information processing, assessment and distribution (Robert Koch-Institut 2019: 8–12; 17; 25; 39). One example is the WHO mission to China to investigate the outbreak and containment of SARS-CoV-2 in February (World Health Organization 2020). This outlined international framework defines the institutional context of the German Robert Koch Institute (= RKI). The Infection Protection Act (§4 IfSG) assigns the RKI the task of supporting political decision-making within the framework of prevention and control of communicable diseases through its own research. In the course of the pandemic, the RKI’s expertise has played a key role in the judicial proportionality review. This will be discussed further below.

So how do science and the principle of proportionality come together? In accordance with the International Health Regulations, the principle of proportionality applies to both international and national pandemic planning when it comes to reducing the mortality and morbidity of a virus through NPIs (Robert Koch-Institut: 19; World Health Organization 2019: 10). Measures that make sense from an “epidemiological point of view” can “result in negative societal or economic consequences”—the “balancing of legal interests” is required (Robert Koch-Institut 2019: 19–20). The pandemic planning of both the WHO and the Robert Koch Institute consists of a scientific part and a recommendation part. In its evaluation of NPIs, the WHO working group uses GRADE, a method derived from evidence-based medicine (“Grading of Recommendations Assessment, Development and Evaluation”, Schünemann et al. 2011; World Health Organization 2019: 11–12). Meta-studies of randomized controlled trials are considered the gold standard of scientific evidence, followed—in descending order—by observational studies, then simulation studies. The lowest level of evidence is provided by expert opinions. The quality hierarchy is based on the motivation to exclude methodological biases as far as possible, like, for example, confounding effects in the material or subjective opinions of the researchers themselves. This is important because expert opinions have played an overwhelming role in the pandemic—in court, in political deliberations and in the media. GRADE now combines evidence—facts—with recommendation—normativity; a particular intervention may have low to moderate evidence and low effectiveness when measured against the standard of randomized controlled trials and *still* be recommended because the costs are low, or the pandemic may be an exceptionally severe one (World Health Organization 2019: 2; 12). Workplace closures, for example, are recommended by the WHO as the last step of work-related measures in exceptionally severe epidemics. Workplace closures can be effective, but the evidence is limited because there are only simulation studies on the subject. Due to the serious economic consequences, the measure would probably only be accepted if there were job and income security for workers (World Health Organization 2019: 54–57). Another example: the WHO recommends school closures conditionally. Their effectiveness is contingent and depends, among other things, on timing, while at the same time they have negative health and socioeconomic effects for both children and parents, for example due to the childcare burden imposed (World Health Organization 2019: 50–52).

In short, neither a specific level of evidence nor a specific virus gives recommendations on its own. Rather, GRADE embodies generalized proportionality considerations, the specification of which is the responsibility of subsidiary nation-state entities. During the Ebola outbreak in 2014, Sierra Leone imposed a 3-day lockdown in Free Town, Port Loko and Kambia. Liberia temporarily imposed a curfew on the capital Monrovia. These measures were described as ineffective and counterproductive because Ebola spreads symptomatically, and the mass quarantine limited health care on the ground (Coltart et al. 2017). Large-scale quarantine measures in Toronto/Canada during the SARS-1 outbreak 2003 have been criticized as similarly ineffective (Schabas 2004). This suggests that non-pharmaceutical interventions are not simply about the application of science, but about politics. The International Health Regulations are characterized by structural non-compliance on the part of the member states and the WHO does not have the authority to enforce compliance (Gostin and Sridhar 2014: 1734).

However, the apparent assumption that decisions on restrictive measures are made solely for reasons of political power does not apply. Political decision-making processes are more likely to be legitimized by scientific knowledge. There is talk of a knowledge society in which evidence-based legislation has become part of the social structures of expectation (Ismer and Meßerschmidt 2016: 91). At the same time, policy is part of a general cultural trend toward prevention and precaution. Since the 2000s, the consideration of the precautionary principle in decisions under uncertainty has been discussed in the field of global health governance (Martuzzi and Tickner 2004). However, there is disagreement about the required level of scientific certainty (Foster et al. 2000). Acting according to the precautionary principle is about avoiding certain risks where both the extent and the underlying causal relationships are not fully understood. One example are genetically modified organisms, whose effect on human health has repeatedly been the subject of controversy (Meßerschmidt 2020: 272). The causal links are not clear, and policymakers are left with discretionary powers in the approval of certain genetically modified products for reasons of safety and prevention. There have been repeated lawsuits before the European Court of Justice over attempts by nation states to ban certain products approved at EU level on their territory (Guida 2021).

With respect to the prevention of infectious diseases, governmental authorities such as the Robert Koch Institute have to take action already in the run-up to a disease and not only when a corresponding risk is realized (Hollo 2020: 31). What is seen as an acceptable risk in each case is not only related to objective criteria such as the extent, reversibility or available options for action, but also to the public perception of risk. There can be feedback effects: the Robert Koch Institute is legally obliged to communicate to the public in order to achieve acceptance for certain interventions such as social distancing. In assessing the severity of an epidemic, the Institute has to take the public's perception into account (Robert Koch-Institut 2019: 3). On the other hand, pandemic reporting may increase risk-perception among the public (Klemm et al. 2016). Accordingly, it may be that NPIs are subject to the calculations of political interests. However, interests, for their part, are articulated only in a historically longer path of powerful cultural ideas, as can be said with Max Weber (Eastwood 2005).

In summary, the functioning of global health governance can be characterized by three intertwined principles: the proportionality principle, scientific evidence, and the precautionary principle. Global health governance is part of a knowledge society; at the same time, the requirements for certain knowledge are not clearly defined. This arrangement is—paradoxically—characterized not by clarity, but ambiguity. In the proportionality assessment of non-pharmaceutical intervention measures, facts and normativity meet, but facts are contested, and norms—such as risk and hazard acceptance—are likewise not objective, but subject to social change. In this context, not only political actors have to make decisions, but also the actors of law—the courts. The following section is devoted to the interplay of law and politics in their access to facts.

### Evidence, Law and Politics

Facts are important for judicial decisions—for example, in the case of violations of traffic regulations—without necessarily treading on scientific terrain. The state may not place itself in any arbitrary relationship to questions of fact, but at the same time it has a constitutionally granted discretion that allows selective access to knowledge (Meßerschmidt 2020: 276–277; Steinbach 2015). The more uncertain the facts, the greater the discretion. In turn, the discretion narrows with the depth of the encroachment on fundamental rights, and the burden of proof increases (Meßerschmidt 2020: 283–284; Steinbach 2015). Conversely, the courts are expected to exercise judicial restraint for reasons of democratic legitimacy (van Gestel and Porter 2016: 176–177). The judicial evaluation of political decision-making processes is then sometimes seen by the public as an intervention in political processes rather than as a review (Popelier and Jaegere 2016: 188). Accordingly, an aspect considered by the courts is the public acceptance of their decisions (Popelier et al. 2021: 2–3). The density of legal control varies; the relationship between the state and the judiciary is not a fixed one. Density of control means the strictness of the standard with which a court subjects the assumptions underlying a law or a regulation to review. The scrutiny can be a rather rough and commonsense-based examination, but it can also be an extensive assessment of the knowledge used by the political decision maker, up to the point of aiming for the best possible balance between the legal rights to be restricted and those to be protected.

The judicial situation gets further complicated by its relationship to science. The Daubert standard is an attempt to make criteria of scientificity the criterion for admitting expertise to court (Cheng and Yoon 2005: 477). While useful in itself, the Daubert test is perceived as a criterion that is too imprecise for practice (van Gestel and Poorter 2016: 179). However, the Daubert standard is not a 1:1 translation of the scientific evidence hierarchy. Regardless of the acceptance or rejection of the Daubert standard, scientific knowledge production during the Corona pandemic is characterized by a polarization which is typical of crises situations (Angeli et al. 2021; Brownlee and Lenzer 2021). Epistemic communities have formed in which questions of knowledge and values are intertwined. In science research crises are regarded as high-cost situations in which science communication is subject to politicization and epistemic conflicts are carried out in the mass media (Rainey et al. 2021). If courts, for their part, now have to make high-stake decisions in weighing up legal interests—freedom or life—the judgment of proportionality is politicized toward *both* the normative and the empirical side.

Likewise, the legal basis for pandemic policy since 2000—the German Infection Protection Act (“Gesetz zur Neuordnung seuchenrechtlicher Vorschriften”)—does not provide clarity in the relationship between politics and law, but rather reproduces the discretionary space in which politics and law operate. Indeed, the Infection Protection Act is subject to the principle of proportionality (Kießling 2020: 258). At the same time, however, with the Infection Protection Act in 2000—long before the current pandemic—the legislature created a so-called “general clause” of epidemic control in §28 (“Protective Measures”), which does not prescribe or limit the executive to certain measures, but instead allows the “necessary protective measures […] to the extent and for as long as necessary to prevent the spread of communicable diseases”. The legislature created the general clause with the intention of being able to take appropriate measures for the emergence of unforeseeable cases—here again in alignment with a cultural trend line oriented toward precaution. Furthermore, the talk of “necessary” actions again points to the principle of proportionality. In the course of the pandemic, the Infection Protection Act has been modified repeatedly since March 27, 2020 by the German parliament, the Bundestag. The spring and autumn interventions were executive in nature and invoked the general clause of §28 IfSG. The intervention policy worked by means of decrees. The general clause was found to be increasingly insufficient and was only revised *after* the second lockdown decision of October 28, 2020 on November 18, 2020, when Parliament passed the Third Population Protection Act. Only now were measures like business closures, mask mandates and curfews part of a catalog of measures. Until then, the executive—in the form of informal agreements between the governments at the federal and state level (“Bund-Länder-Konferenz”)—was gaining more and more de facto importance. The executive interventions took the form of law ex post—the legislature was thus always one step behind. The Third Population Protection Act explicitly made individual freedom rights—in occupation, movement, education—contingent on the infection control (Bundestag 2020: 2402). This reflected the political infection suppression strategy that had been pursued since spring 2020. As will be discussed below in further detail, the fundamental rights to life and health have formally been given legal priority status—at least from a political point of view.

The legal assessment of the legislative and regulatory practice is a matter for the courts. So again, what is proportional? In the principle of proportionality, normative evaluations and empirical facts come together, but these empirical facts are not unambiguous, they are disputed. And precisely because there is judicial discretion in the density of control, the judicial determination is a political determination. Any judicial judgment on the proportionality of non-pharmaceutical interventions thus enters the political arena. Abstract considerations do not help at this point. Understanding the proportionality test as a negotiation arena between law, politics, and science, I will turn to an analysis of selected court decisions during the Corona pandemic in the following section.

### Scientific Evidence and Proportionality in COVID Jurisprudence

In its annual report published in March 2021, the German Federal Constitutional Court lists 880 proceedings related to SARS-CoV-2. Besides the rejection on formal grounds, many appeals have been rejected on the grounds of the state's constitutional duty to protect life and physical integrity. According to the report, the dangers to life and health outweighed the restrictions on liberty in each case (Bundesverfassungsgericht 2021a: 59–60). This was also the case with the Federal Constitutional Court's ruling on school closures—analyzed below—issued in November 2021. The situation was similar at the level of the administrative courts. For this, I refer to two quantitative studies from the legal sciences—the only ones I know of for German jurisdiction (Klafki 2021; Kruse and Langner 2021). I briefly review those aspects of both studies that are relevant at this point.

At the beginning of the pandemic, the courts used legal argumentations to support the political interventions. In case of doubt, the courts would have voted for protection—they did not want to be responsible for the overwhelming of the health system in March 2020 (Klafki 2021: 595). The “principle of proportionality” is “as a formal principle of consideration, in principle flexibly adaptable to actual circumstances”. (Klafki 2021: 595). It was only with the increasing duration of the pandemic that the judicial control density of politics had increased, and the success rate of lawsuits had risen over the summer until October 2020. However, Klafki's study is methodologically limited by the fact that it only covers the period until the end of October 2020. It was not until October 28, however, that politicians decided to close restaurants, hotels and leisure facilities, before also closing the retail sector on December 16. Rulings on lawsuits against the closures could no longer find their way into the study. Authors Kruse and Langner used text mining to examine administrative court rulings between March 1, 2020 and September 30, 2021. They conclude that the average “liberty rate”—the success rate percentage in favor of liberty rights—remained at the same level (18%) during the pandemic as before the pandemic. Quantitatively, this may be true, but not all rates are the same. The same freedom quota is on a qualitatively different level when bars, theaters and schools remain closed, but at the same time lawsuits against the restriction of the right of assembly are successful. These figures need to be interpreted. Following Klafki: The *form* of the proportionality test is one thing, its *substantial* negotiation in the concrete case is another. With that, I turn to the case analysis.

I will analyze three cases that were decided on three different points in time: the first pandemic wave in spring 2020, the second wave in autumn 2020 and the start of the third wave in spring 2021. All cases have two things in common: The subject matter of their judgment falls in each case into a period of a perceived acute danger in which political actors felt compelled to engage in restrictive interventions to prevent an overwhelming of the health care system.

Few methodological remarks on the criteria for case selection: all cases can be found in “Juris.de”,^3^ a German-language database for the compilation of court rulings, statutory provisions and legal commentaries in professional journals. The selection of cases examined here is an attempt to capture the characteristics of the events. Three criteria are of importance: timing, the nature of the proceedings and public interest.

1. Firstly, it is to be expected—in accordance with the principle of proportionality—that the density of judicial control will increase with the increasing duration of the interventions. The legal scholar Andrea Klafki believes that this is precisely what happened in the period between March 2020 and October 2020. I have therefore picked out three key events during the pandemic, each separated by half a year. These events are each characterized by a surging incidence of infection, on the basis of which policymakers have made decisions to intervene. If the density of judicial control had increased over time, this should become evident in the November ruling after the autumn lockdown on October 28, 2020.
2. Secondly, another point: The court rulings from the months of March, November and October 2020 are rulings in applications for interim relief. This form of litigation was the typical court case in the pandemic due to the time requirements. The proportionality test is comparatively less comprehensive here – although, at the same time, German legal scholars observe that summary proceedings are also capable of a differentiated review of the situation (Schmitt 2021: 471). The Federal Constitutional Court ruling of November 19, 2021 on the so-called Fourth Population Protection Act from April 2021, on the other hand, is a main proceedings. Here, there is more time and thus the possibility of a thorough assessment of the facts. A comparatively high level of control is to be expected here.
3. Other selection criteria are public interest and controversy. School closures were already very controversial in spring 2020, and many European countries (Spain, Switzerland, Sweden, and England) had much shorter periods during which children were not taught in attendance while facing the same problem—a pandemic. Moreover, the effectiveness of school closures was and is disputed within the scientific community, but the burden on the children accumulated over time.

The case selection is thereby meant to exemplify what I call an arena of negotiation between law, politics, and science. The following questions guide the analysis accordingly:

- How do politics justify its decision?
- What expertise does the judicial proportionality test draw on—and what does it leave out?
- What is the relationship between the evidence used (= facts) and the balance between the legal interests (= normativity)—and how does this relate to the political dimension?
- To what extent do the judicial proportionality tests resort to formulations of the precautionary principle—and what standard of certainty is required?

On the one hand, these questions reflect the potential politicization of science in courts. The mode of political precautionary action is reflected in each case in the judicial proportionality test in specific semantic forms like epistemic uncertainty, of preventing a threat to the life and health of the population, and of the discretion granted to politics. On the other hand, these questions also reflect the variable control density of courts: the higher the judicial standards of evidence, the higher the density of control. The depth of the fundamental rights restrictions makes it to be expected that courts will climb the hierarchy of evidence parallel to the increasing duration of the pandemic.

### March 2020

Most of the restrictive measures imposed in Germany—event bans, school and business closures—took place between March 9 and March 22 in response to exponentially rising infection rates. There were differences in speed between the German states. The Bavarian government was a forerunner nationwide and, even before the decision at the federal level, closed businesses by decree on March 16 in response to rising infection figures in order to reduce the burden on the health system (Bayerische Staatsministerien für Gesundheit und Pflege sowie für Familie, Arbeit und Soziales 2020: 2). In the public notice, the “temporary ban order” is claimed to be explicitly “proportionate.” (Bayerische Staatsministerien für Gesundheit und Pflege sowie für Familie, Arbeit und Soziales 2020: 2).

On March 18, 2020, a shop owner filed a lawsuit against the closure of his business before the Administrative Court of Munich (Verwaltungsgericht München 2020). In its rejection, the court justified the proportionality of the business closures with a wording characteristic of the precautionary principle, such as the threat to life and health (Verwaltungsgericht München 2020: para. 25) and epistemic uncertainty.

Regarding the effectiveness and necessity of the measures—the first and second step of the proportionality test, the court stated, drastic measures are necessary to prevent a collapse of the health care system, milder means of social distancing while the business is open are not realistic (Verwaltungsgericht München 2020: para. 28). At the same time the court stated, the effectiveness of the measures taken had “admittedly not been conclusively scientifically investigated” (Verwaltungsgericht München 2020: para. 26). Ultimately, however, in face of the impending danger, “the public interest in physical integrity” outweighs the “private, predominantly economic interests of the applicant.” (Verwaltungsgericht München 2020: para. 28).

In its assessment of the effectiveness and necessity of measures taken the Administrative Court refers to a Modeling Study of the Imperial College London COVID-19 Response Team, which, according to the court, suggests that workplace closures are a necessary and a required “building block in slowing down the infections” (Verwaltungsgericht München 2020: para. 26). The study carried out for the USA and the UK appears to be “quite transferable to Germany” (Verwaltungsgericht München 2020: para 26). According to the science journal “Nature”, Report No. 9 was the most cited preprint of the year 2020 (Callaway et al. 2020). Measured against this fact, the court's decision has a comprehensible rationality.

Measured against the standards of scientific evidence, model calculations such as those of Report No. 9 can claim a comparatively low level of evidence (Mansnerus 2013). The predictive use of models for the spread of infectious diseases depends on the completeness of knowledge about aspects such as population heterogeneity, transmission routes and climatic factors (Keeling/Rohani 2008: pp. 8–10). In its risk and strategy recommendation (“high”, Verwaltungsgericht München 2020: para. 25; “to delay the further spread of the virus as far as possible”, Verwaltungsgericht München 2020: para. 25) the court refers to the RKI, a federal agency which is bound by political directives. In order to assess the adequacy of certain measures, the court relied on a model that had also been well received within RKI simulations. The Imperial College modeling was itself an integral part of “SAGE”, the official government “Scientific Advisory Group for Emergencies” in the UK. Counterfactually, assuming a higher density of control, the court could have demanded better evidence given the intensity of the intervention. For example, on March 20, the German Network for Evidence-Based Medicine criticized non-evidence-based measures (Deutsches Netzwerk Evidenzbasierte Medizin 2020a). Hospital hygienists proposed an alternative risk-stratified approach early in March which referred to the data known until then on the risk distribution for mortality and hospitalization (Walger et al. 2020). However, the scientific quality of evidence is not necessarily the politically decisive element.

The political decision-making situation was characterized by uncertainties about the future: first, uncertainties about the effectiveness of measures and their societal harms; then, second, uncertainties about the development of infection and mortality rates if stores were not closed. However, as Klafki (see above) points out, in this situation of acute danger and uncertainty, the courts were keen to support interventionist policies—the control density was accordingly low. It is consistent with this political orientation of the court that the judicial proportionality review invokes the same expertise as the policymakers themselves. With increasing duration, the burden of proof for interventions would have to increase, and the density of judicial control would have to tighten. A test of this assumption are the judicial reviews of political decrees in autumn 2020 at the beginning of the second pandemic wave in Europe. The following section deals with two exemplary cases.

### October/November 2020

On October 28, the federal and state governments decided to close hotels, restaurants, gyms, and cultural institutions in reaction to renewed increases in the number of infections to avoid the threat of overwhelming the health care system and the deaths that would result. Because retail remained open, it was referred to as “Lockdown Light”. Politicians justified their action with the ignorance of 75% of all infection sources. Full tracing of infections would have to be ensured again in order to keep the virus spread under control. These measures were held to be “necessary” and “proportionate.” (Bund-Länder 2020a: 5). In her speech in the German Bundestag, Chancellor Angela Merkel even affirmed the proportionality formula twice: “Therefore, I repeat: the measures we are taking now are adequate, necessary and proportionate.” She ruled out the possibility of a strategy focused on protecting at-risk groups. The syntax used suggests that the Chancellor was well aware of the legal dimension. Nevertheless, lawsuits followed swiftly, but the courts upheld the policy line.

On November 4, 2020, the Higher Administrative Court of Saxony-Anhalt justified the rejection of a lawsuit against the closure of hotel establishments with the “considerable uncertainties” (Oberverwaltungsgericht des Landes Sachsen-Anhalt 2020a: para. 70) that it was “not ruled out” that “virus transmissions occur in accommodation establishments after all.” (Oberverwaltungsgericht des Landes Sachsen-Anhalt 2020a: para. 88) The greater the possible damage, the lower the requirements “for the probability of the occurrence of damage” – and thus also “the requirements for proportionality”, and precisely this “applies in the present case” of an infection with SARS-CoV-2 (Oberverwaltungsgericht des Landes Sachsen-Anhalt 2020a: para. 77). The court recognized “a serious risk situation that requires state intervention […] to prevent an exponential growth of infections with immediate, unforeseeable consequences for the health, life and limb of the population […]” (Oberverwaltungsgericht des Landes Sachsen-Anhalt 2020a: para. 76).

With respect to the effectiveness of the measures – the first part of the proportionality test –, the court stated, on the one hand, “accommodation establishments are not among the drivers of the pandemic”, but, on the other hand, “the circumstances of infection […] were unclear in the meantime in more than 75% of cases” (Oberverwaltungsgericht des Landes Sachsen-Anhalt 2020a: para. 88). In other words, it could not be ruled out that accommodation establishments were not drivers of the pandemic after all. Furthermore, the ban on accommodations also indirectly served to prevent the spread of the virus to the region by people traveling to the area. The second part of the proportionality test assesses the necessity of the measure: Less stringent measures, such as a strategy aimed at protecting at-risk groups, would not work because younger people are likewise potentially affected and possible long-term consequences are still unclear, the court said (Oberverwaltungsgericht des Landes Sachsen-Anhalt 2020a: para. 80). In the weighing of interests—the last step of the proportionality test—between the right to life and physical integrity on the one hand, and the property rights and freedom of occupational choice—which are affected here—on the other hand, “the protection of life and physical integrity prevails” (Oberverwaltungsgericht des Landes Sachsen-Anhalt 2020a: para. 115).

On October 27, just one day *before* the “Lockdown Light”-decision on the federal level, the same Higher Administrative Court of Saxony-Anhalt upheld the appeal against a de facto ban on accommodation (Oberverwaltungsgericht des Landes Sachsen-Anhalt 2020b). The complaint was against the state decree according to which travelers from infection risk areas had to present a PCR test that was negative for no more than 48 h. The owner's main clientele came from these risk areas. The regulation therefore appeared to the applicant to be a de facto occupational ban (Oberverwaltungsgericht des Landes Sachsen-Anhalt 2020b: para. 7). The court made almost the same assessment as a week later (Oberverwaltungsgericht des Landes Sachsen-Anhalt 2020b: para. 25). With regard to the first and second step of the proportionality test, the measures may be effective in containing the virus, and there may be no milder means of preventing the spread of the virus (Oberverwaltungsgericht des Landes Sachsen-Anhalt 2020b: para. 27). However, the measure is not proportionate in the narrower sense—that is, at the third stage of the proportionality test. The Court states that “accommodation establishments are […] still not among the drivers of infections”. (Oberverwaltungsgericht des Landes Sachsen-Anhalt 2020b: para. 36) The state government is “obliged to ensure proportionality” and must therefore “continuously and in a differentiated manner examine whether concrete infringements of fundamental rights are still reasonable.” (Oberverwaltungsgericht des Landes Sachsen-Anhalt 2020b: para. 33). With “increasing duration”, measures would have to be reviewed “particularly rigorously.” (Oberverwaltungsgericht des Landes Sachsen-Anhalt 2020b: para. 33). Since the government continues to keep bars and sports facilities open, it implicitly accepts a certain risk to life and health (Oberverwaltungsgericht des Landes Sachsen-Anhalt 2020b: para. 34). However, the state government had not been able to show “that there is a particularly high risk of infection in connection with the provision of accommodation”. (Oberverwaltungsgericht des Landes Sachsen-Anhalt 2020b: para. 36) The state government “refers to increasing infection figures in general, without taking the place of infection into consideration.” (Oberverwaltungsgericht des Landes Sachsen-Anhalt 2020b: para. 38).

The juxtaposition of the two judgments is important for the analysis of judicial proportionality review for several reasons:

1. In her empirical analysis—see above—Klafki (2021) observed an increasing control density of the courts over time. The October ruling of the Higher Administrative Court of Saxony-Anhalt proves her right. However, Klafki's analysis only covers the timespan from March to the end of October. *After* the lockdown decision, things look different again: with unchanged infection dynamics, one and the same court comes to a different proportionality judgment, although both rulings explicitly and repeatedly grant policymakers broad discretion in averting danger (Oberverwaltungsgericht des Landes Sachsen-Anhalt 2020b: para. 12; 20; 24; 33; Oberverwaltungsgericht des Landes Sachsen-Anhalt 2020a: para. 70; 76; 79; 93; 100).
2. The courts may use a typical wording of the precautionary principle in each case, but the balancing of the interests to be protected – the third step in the proportionality test – turns out differently, in favor of the executive branch. In the November ruling, the interference with the right to freedom of occupation and property rights to be justified was even somewhat higher than in the October ruling. The decisive factor for the court's tendency here seems to be the decision at the federal level, while decisions at the state level have tended to be overturned until then. This corresponds to the growing importance of the federal level during the pandemic compared to the originally—according to §32 of the Infection Protection Act – state level organized disease control since March 2020, since Chancellor Merkel chaired the federal-state consultations.

This leads to another aspect that renders the present court decision exemplary. In the proportionality test, facts and normativity meet—so how does it relate to the use of scientific knowledge in court in the present case? In the risk assessment as well as in the presentation of the existing knowledge and non-knowledge about infections, the court refers to the publicly available data of the RKI. The RKI remains the only scientific source of information for both court decisions. The November ruling even copies almost word for word the entry on the Robert Koch Institute's website about the severity of the disease, age distribution, and possible long-term consequences (Oberverwaltungsgericht des Landes Sachsen-Anhalt 2020a: para. 74). This aspect is of importance for several reasons:

1. First, while in the October ruling the court shifted the burden of proof to justify interventions to the legislature on the grounds that an increasing duration of interventions requires greater justification by facts, the November ruling shifts the burden of proof within only one week: it is not evidence for effectiveness that is required, but evidence for the absence of danger which is required. The same court made diametrically opposed decisions on the proportionality of business closures within a week though the evidence did not change. The reported infection numbers had been rising continuously since September, and the health authorities were often no longer able to trace infections. The identical knowledge mobilized by the court to justify its proportionality assessment is knowledge about ignorance, which did not legitimize intrusions into individual freedom and private property in October, but did legitimize an intrusion into individual freedom and private property in November. In its access to facts, the court adopts the normative judgment of the federal policy.
2. Second, the RKI is not – as legal scholar critics mentioned from time to time – an independent institution. The RKI is a federal authority within the remit of the Federal Ministry of Health and is bound by its political directives. The head of the institute is proposed by the Federal Minister of Health and elected by the Federal Government. The Infection Protection Act (§4 IfSG) assigns the RKI the task of supporting political decision-making within the framework of prevention and control of communicable diseases through its own research. The Robert Koch Institute risk assessment cited by the courts, for example, is an explicitly politicized assessment insofar as it must take into account societal values and societal risk acceptance (Schmitt 2021: 472). It is not a risk assessment based solely on objective statistics. Nevertheless, courts routinely refer to the RKI's expertise in their judgments (Kruse and Langner 2021: 3711–3712). The present case is not a non-representative outlier.
3. Third and furthermore, with regard to decision-making, the Robert Koch Institute supported the political pandemic control strategy by publishing its own strategy papers, which in turn formed a mixture of normativity and facts. An example relevant here: On March 4 and March 19, 2020, the RKI published strategy papers. There was general talk of social distancing and the cancelation of major events and school closures, but not of workplace closures across the board. The RKI reckoned that at a certain point the spread of the virus could no longer be stopped (“community transmission”) and that not all cases could be traced, so that resources would have to be focused to an increasing extent on protecting at-risk groups. After the first lockdown on March 23 politicians adapted an infection suppression strategy as early as spring 2020 on their way out of the first lockdown: the goal now was to “protect all people in Germany as well as possible from infection” (Bund-Länder 2020b: 2). New infections should be kept as low as possible and fully traced. On October 23, 2020—In the midst of the next infection wave. The RKI now officially recommended an infection suppression strategy in a paper dated October 23, 2020, in which the aim was to “minimize the spread and negative health ramifications of the pandemic” (Robert Koch-Institut 2020: 2). Only under the proviso of infection protection the Robert Koch Institute did caution that the “measures should be legally and organizationally proportionate” (Robert Koch-Institut 2020: 2). Finally, on November 18, 2020, the German Bundestag passed the Third Population Protection Act, a revision of the Infection Protection Act, among other things. The Third Population Protection Act was in line with the political strategy pursued until then and explicitly made individual freedom rights—in occupation, movement, education—contingent on low infection numbers (Bundestag 2020: 2402).

From a certain point of view, Jurisprudence remained within the framework of the health policy defined in October as well as in November 2020. At the same time the courts justified the proportionality of measures by reference to a public health agency that is also legally bound to government policy. This is perhaps the most obvious form of politicization of the judicial proportionality test. As discussed above, the Court draws on formulations of the precautionary principle in both proportionality tests, but these are insufficient to explain the substantive change in the proportionality test within a week.

Under the counterfactual assumption of a higher control density and after more than half a year into the pandemic in Germany, courts could have questioned the scientific independence of a health authority subordinate to the state and demand independent and stronger evidence. The German Network for Evidence-Based Medicine, for example, stated at that time that there was a lack of scientific evidence for the effectiveness of interventions, because there was no control group that had proceeded without interventions (Deutsches Netzwerk für Evidenzbasierte Medizin 2020b). On the other hand, since spring 2020, a group of authors around the health economist Matthias Schrappe has been promoting the idea of an alternative pandemic strategy: measures should be differentiated according to risk stratification by age and pre-existing disease. Not infections at all, but certain high-risk infections should be avoided. Country comparisons would also suggest that there are milder means of social distancing instead of the restrictive interventions taken so far (Schrappe et al. 2020). The proposals were surprisingly similar to the original RKI strategy from March 2020.

Up to this point, I have analyzed applications for interim legal protection, so-called summary proceedings, at administrative courts, whose proportionality test is less complex than main proceedings due to the time requirements. As a third case, the analysis therefore turns to a main proceedings before the highest German court, the Federal Constitutional Court, on one of the most controversial interventions during the pandemic—the school closures during the third wave of the pandemic in spring 2021.

### April/November 2021

On April 22, 2021, the “Fourth Law for the Protection of the Population in the Event of an Epidemic Situation of National Significance” came into force, after the German parliament had passed the law only one day before. Part of this law was the reform of the Infection Protection Act: §28b IfSG regulates curfews and school closures nationwide. Both measures were controversial; nevertheless, for reasons of limited space and material, I will focus on school closures. A parents' initiative that filed the lawsuit has made the documents surrounding the trial publicly available on a website. The scientific expert opinions can also be found there.^4^ The rule was the following: if a district or an urban municipality exceeded the incidence of 165 per 100,000 inhabitants for three consecutive days, school attendance was canceled.

The federal government justified the bill with renewed increases in the number of infections due to mutations of the coronavirus. According to the government fractions, the target was "to slow down the further spread of the virus and to break the exponential growth in order to avoid an overload of the health care system as a whole and to ensure medical care nationwide." (Bundestag 2021: 8) The parliamentary groups explicitly referred to the state's constitutional “duty to protect the fundamental right to life and physical integrity” as well as the further goal of “ensuring the functioning of the health care system as a paramount common good”. (Bundestag 2021: 8) School closures—along with curfews—are an intensive intervention, but also particularly effective, they therefore lead to a faster “return to conditions with as few restrictions as possible”—which contributes “especially to their proportionality”. (Bundestag 2021: 11). The wording fits into the scheme of political precautionary action, where actors are aware of proportionality concerns. With this law, the federal government took away a part of the states' competence in pandemic control. In public, the federal government judged the measures taken at the state level to be insufficient.

Lawsuits against federal laws go to the Federal Constitutional Court. They do not take the route through the administrative courts. In the following, I will not deal with the rejected judgment in the summary proceedings, but with the main proceedings. The judgment in the main proceedings was issued on November 19, 2021. What strikes as special now is that half a year passed between November 2020 and April 2021, and another half a year passed between April 2021 and November 2021. This plus the formal fact that it was about main proceedings lead us to expect a correspondingly higher level of control density.

In April 2021, the court said in its ruling, exponentially rising infection rates were a danger "to life and limb" and threatened the functioning of "the health system" (Bundesverfassungsgericht 2021b: para. 156). According to the Federal Constitutional Court, school closures were proportional, meaning they were “adequate”, “necessary” and “appropriate” (Bundesverfassungsgericht 2021b: para. 109). Regarding the first step of the proportional test, the effectiveness assessment focused on the infectiousness of children and the associated causal influence of school closures on the course of infection in the overall population. A majority of the expert opinions quoted assumed an age-dependent infectiousness: the younger, the less infectious (Bundesverfassungsgericht 2021b: para. 117). Two of the opinions took the related view that schools were not “’drivers’ of infection rates”’ (Bundesverfassungsgericht 2021b: para. 118). A minority opinion held that children were less infectious but contributed as much to the infection rates as adults because of their many social contacts (Bundesverfassungsgericht 2021b: para. 117). The court concluded – maybe surprisingly – that it was therefore “reasonable” to assume that open schools “contribute to the infection-related risk to life and limb of the population” (Bundesverfassungsgericht 2021b: para. 118). Regarding the necessity of school closures, according to the court, there was a lack of knowledge about measures with a more targeted effect (Bundesverfassungsgericht 2021b: para. 132), and at the same time there was an urgent need for comprehensive measures to protect legal interests of paramount importance (Bundesverfassungsgericht 2021b: para. 17). Additional infections could be prevented with certainty only by closing schools (Bundesverfassungsgericht 2021b: para. 129). It had therefore been justified to exclude all possible risks of infection. School closures—like curfews—should not be considered in isolation, but as part of an overall concept to combat the pandemic. Regarding the third step of the proportionality test, in the final balancing of interests, on the one hand, the court acknowledged that distance education could not provide the same functions as face-to-face teaching, and that the periods of distance education accumulated over the period of the pandemic had significant consequences for the development of the children's personalities and thus violated fundamental rights (Bundesverfassungsgericht 2021b: para: 17). On the other hand, in the balancing of the children's right to education and the protection of life and health, the court ruled that the school closures were ultimately also “proportionate in the narrower sense”, serving “public interests of overriding importance” (Bundesverfassungsgericht 2021b: para. 133).

In the proportionality test, weighing the effectiveness and consequential costs of particular acts, the court confirms the political situation assessment of April 2021. But first of all, it should be noted that the Federal Constitutional Court's ruling, measured by its comprehensiveness and the number of scientific expert opinions consulted, exhibits a high level of control density. The majority opinion on the infectivity of children comes from professional societies (German Society for Paediatric Infectious Diseases, but also the Robert Koch-Institute) or university institutions (COVID-19 Data Analysis Group LMU Munich). The minority opinion was held by the internationally reputated virologist and government advisor Christian Drosten, who since 2020 has advocated in scientific publications as well as in public the hypothesis that children are just as infectious as adults. He has also repeatedly defended school closures accordingly.

But science does not work according to the majority principle, as a judicial selection of expert opinions might suggest. It is more complicated. However, *within* the scientific community, there have been studies on the infectiousness of children, some of which have been diametrically opposed to each other, but each of which has also been methodologically different. A meta-study based on evidence criteria finds methodological uncertainty and inconsistencies in the data on school closures. (Talic et al. 2021: 7–8). Two examples from the scientific journals: a case study at a Belgian school believes to prove that children are equally infectious as adults (Meuris et al. 2021). A Japanese study, on the other hand, believes that no causal effect of school closures can be proven in a comparison of prefectures (Fukumoto et al. 2021). The latter study may be higher on the evidence hierarchy than a case study due to its method of comparison—but how certain is the result; and what certainty does it require?

With regard to the constitutional court's proportionality test, the lack of certain knowledge about the effectiveness of measures given an unspecified risk of infection through interpersonal contact was sufficient for the court to justify the interventions. The plausibility of possible effects was sufficient before the court in the face of a pandemic situation that was assessed as dangerous. The court-based plausibility of the possible effectiveness of school closures predominantly on the minority opinion of a scientist who not only advises government policy but is also not unbiased toward the measures. Under the counterfactual assumption that the control density of the political decision-making process would be all the higher in view of increasing duration and restrictiveness, the Federal Constitutional Court could have drawn different conclusions in light of the not entirely certain expert opinions—both with regard to the necessity of school closures and the balancing of the protected interests of life and education.

While the Federal Constitutional Court referred to experiences in neighboring countries in its proportionality test of the curfew—not analyzed here –, the ruling on school closures left out the positive comparison with countries that never closed their schools—like Sweden—or only for a short time—like Switzerland and England. Even countries like France or Spain, which in part and at the beginning of the pandemic took harsher measures than Germany, hardly closed schools after the first wave, if at all.^5^ The court also did not consider a differentiated solution according to the age of the children—for example, keeping primary schools open—to be possible, although this would have been suggested by the majority of the expert opinions. In the selection and interpretation of the expert opinions, the court instead opted for the greatest possible exclusion of infection risks, assuming the worst-case scenario. The court's wording—on epistemic uncertainties and the danger to life and health to be prevented—suggests thinking in the precautionary mode. However, the precautionary principle—as mentioned above—does not of itself determine a particular standard of scientific safety. It therefore seems more plausible that the courts made a political judgment in the proportionality test.

## Summary of the Judicial Proportionality Test

The proportionality test can be seen as an arena of negotiation of global health between science, politics and law. So what can be said about the general approach taken by the courts? I will give a brief summary in a few key points.

- With increasing duration comes the expectation of greater control density. Interventions in constitutional rights must be more differentiated, uncertainty about empirical facts is or has to be reduced. Political decision-makers, one could say, are expected to provide stronger justification for interventions into fundamental rights in autumn 2020 than at the beginning of the pandemic in spring 2020, and in spring 2021 this burden of justification increases again.
- The October court ruling corresponds to the expectation of a higher density of control—measures must be more differentiated, the burden of proof under conditions of ignorance lies on the side of state intervention. The expectation of a higher density of control also corresponds to the scope and diversity of scientific expertise in the Federal Constitutional Court's ruling of November 19, 2021 on school closures in April 2021.
- On the other hand: As early as November 2020, the picture of increased control density is reversed again: After the political decision on “Lockdown Light”, one and the same court uses the same knowledge or lack of knowledge about infections to the disadvantage of the plaintiffs. What was considered disproportionate in October was considered proportionate in November. The same applies to the Federal Constitutional Court: Contrary to a majority tendency in the expert opinions for a lower infectivity of children, the judgment confirms the political decision: the knowledge is uncertain, a possible effect of school lockdowns could not be excluded.
- In the proportionality test, the courts repeatedly resort to formulations that are very characteristic of the precautionary principle (e.g., danger to life and health; state obligations to protect; acting under uncertainty). However, the use of formulations of the precautionary principle does not say anything about the required degree of certain knowledge. This is shown by the judgments of autumn 2020 and spring 2021.
- Courts select and interpret expertise in a way that confirms policy—more precisely: federal policy. Expertise that is also active in scientific policy advice to the federal government is comparatively more important. Since spring 2020, agreements and decisions at the federal level have gained in importance over policy at the state level. The policies of individual federal states, on the other hand, seem more contestable.

In sum, courts—the Federal Constitutional Court as well as the administrative courts—adopted the infection control proviso, subordinating the freedom of movement and the right to education to the—above-mentioned—political strategy of infection suppression in its proportionality assessment. Existing epistemic uncertainty is interpreted in the wording of the precautionary principle in favor of political decision-makers.

At this point the case study ends. The following section discusses the importance of the public sphere in understanding both politics and jurisprudence.

### Public Knowledge

The proportionality test brings together normativity and facts, and this is precisely what renders it so suitable an arena for negotiation between science, politics and law. For its part, the selection of facts is not value-neutral, the judicial control density of the political process is variable. The selection and interpretation of the expertise in the cases studied here turned out in favor of the political decision-making process—although other judgments of the proportionality test would have been possible under the counterfactual assumption of available evidence, the depth of interventions and the existence of alternative strategies during the pandemic. The judicial risk and hazard assessment on the one hand and the requirement for evidence justifying the interventions on the other seem to go hand in hand. All in all, the judicial restraint reproduces an infection control proviso of liberties. This means that the exercise of individual freedoms is contingent on the course of infection numbers. *Whenever* there is a wave of infections liberties are at the disposal for the higher valued goods of life and health protection.

Public acceptance of politics may explain judicial restraint. But this insight has to be complemented to account for the judicial selection and interpretation of knowledge during the pandemic. It is about what knowledge is publicly accepted. In short, judges themselves read and listen to the news and develop a sense of public expectations. To illustrate this, I draw on an empirical media study on German news coverage during the Corona pandemic (Maurer et al. 2021).

German coverage consistently considered the measures taken to protect against infection to be either adequate or even insufficient (Maurer et al. 2021: 45). Only a minority of articles considered the measure too harsh. In terms of content, the reporting largely conveyed a research consensus on SARS-CoV-2 (60% “unequivocal”, 26% “rather a consensus”, Maurer et al. 2021: 39). This may suggest that the coverage meant to justify their opinion with the assumed scientific consensus. Normatively, the majority of the media thus represented an infection suppression strategy. This corresponds to the opinion of the recipients, the majority of whom judged the measures to be appropriate or even not yet far-reaching enough (Maurer et al. 2021: 12). The scientific consensus assumed by the media is contrasted by a polarization of the scientific community itself, which—as far as the data suggest—is received asymmetrically in the media. The John Snow Memorandum published on October 15, 2020 (Alwan et al. 2020) was a response to the Great Barrington Declaration published on October 4, 2020 (Kulldorff et al. 2020). The latter proposed a risk-stratified strategy, according to which protection strategies should focus primarily on the elderly and the previously ill. The John Snow Memorandum disputes the suitability of a risk-focused strategy and advocates an infection suppression strategy in which virus spread is kept under control through comprehensive testing. According to an impact study, both statements are initiated by equally reputable experts and can therefore claim similar reputational capital within the scientific community, while the John Snow Memorandum dominates quantitatively in media perception (Ioannidis 2022).

Among the signatories of the John Snow Memorandum is the internationally renowned virologist Christian Drosten, who received quantitatively the greatest attention in the German media—in fact “more media attention […] than all other virologists put together” (Maurer et al. 2021: 39). Drosten, along with Anthony Fauci in the USA, Jérome Salomon in France or Jaap van Dissel in the Netherlands, is an example of an international phenomenon of the personalization of science during the pandemic (van Dooren and Noordegraf 2020: 3). As a specialist in coronaviruses, Drosten has become a scientific advisor to the federal government. He reaches a broad public via the podcast of the public broadcaster NDR, in which Drosten interprets events and assesses scientific studies. The personalization of debates lies in the inherent logic of the media. It makes stories tangible—also for judges, who, of course, have only a limited information capacity. At the same time, the media production of personal authority contradicts the quality criteria of scientific evidence to exclude methodological distortions through subjectivity as far as possible. Courts, however, can hardly ignore this public popularity. Popularity, conversely, draws the scope for the selection and interpretation of scientific knowledge in the process of judicial proportionality review. This makes it very understandable that court rulings have attached great weight to Drosten's expertise.^6^ Drosten's expert opinion, for example, on the infectiousness of children or the appropriateness of school closures, can claim validity as widely known and accepted public knowledge—irrespective of the controversies within the scientific community. Drosten's comments or assessments of models or infection control strategies announced via the media have also been used—albeit with a less prominent role—in the other judgments analyzed here (Verwaltungsgericht München 2020: para. 26; Oberverwaltungsgericht des Landes Sachsen-Anhalt 2020b: para. 83). In its access to knowledge, the judicial proportionality test does not operate in a social vacuum, but rather at an intersection of politics, science and public opinion.

## Conclusion

Proportionality judgments bring together normativity and facts. In a crisis situation in which science is divided, a proportionality judgment is also a political value judgment. Under the conditions of public scrutiny, which favors a certain scientific community, the situation of courts is even more difficult. Judges find themselves in a comparable situation to that of politicians, who assess their options for action in the mirror of public opinion. The public sphere thus becomes a kind of stage on which actors from science, politics and law observe each other and weigh up their room for action. How this results in the scope of legitimate expertise for courts was the subject of this article. The result of my analysis: since March 2020 court rulings confirm and reproduce a political infection control strategy. Characteristic of this political strategy is a balance of interests with a priority of life and health over other liberties, which manifests in repeated revisions of the Infection Protection Act. The exercise of individual freedoms is contingent on the course of infection numbers.

There are topics I could address only marginally. I have placed the COVID jurisprudence in the context of a precautionary culture. This was a premise of my analysis. However, this assumption should be better examined and reviewed, especially with regard to its relationship to other societal values. It remains—for example—unexplained why in law, politics and the public, infection prevention enjoys a relatively high preference compared to individual liberties. It seems that a culture of prevention and precaution—despite or because of its success—does not produce a lower, but rather a higher risk-perception, that confirms—and perhaps stimulates—the need for precaution. This requires further research. Unfortunately, this point can only be touched upon here. The same applies to a debate that still needs to be conducted on the consequences of the emerging shift in the balance between freedom and protection in favor of protection, as it seems to be articulated in COVID jurisprudence and legislation. Regarding the political dimension, the relationship between the executive and the legislature—parliamentary legislation confirmed executive measures with a time lag—could only be touched on in a cursory manner. The same applies to the gradual shift of competences to the federal level. Both are complex topics of debate in German legal studies.

Finally, the methodological limitations of this study should be pointed out. The case analyses claim an exemplary character without being able to fulfill a representative claim in the strict statistical sense. Further, methodologically refined qualitative analyses are necessary, which also specifically look at the international level of COVID court decisions with all its variations.

## Footnotes

1 I would like to thank two anonymous reviewers. Their critique has helped me to improve the paper at hand considerably. I would also like to thank the two legal scholars Andrea Kießling and Oliver Lepsius, who provided me with important knowledge on the principle of proportionality.

2 “Lockdown” is a term as frequently used as it is poorly defined. This paper works with a pragmatic definition of lockdown: it understands lockdown to mean extensive coercive measures of social distancing imposed by a government. Their coercive character distinguishes them from voluntary social distancing measures. The scope is variable and not very clear-cut: Lockdowns range from the closure of nonessential businesses to the suspension of attendance classes, the closure of cultural and recreational institutions, individual sectors of commerce, the imposition of mandatory masks in certain areas, the restriction of gatherings and the imposition of curfews. Schools could stay closed while businesses were already opening; curfews could occur while schools remained open. Sometimes there were regional differences within a country.

3 See https://www.juris.de. Access was provided via the university's internal login.

4 See https://schule-bleibt-offen.de/schriftsaetze/ (Last access 25.06.2022).

5 For international comparisons see https://covid19.uis.unesco.org/global-monitoring-school-closures-covid19/country-dashboard/.

6 A quick search in the data archive Juris.de for court decisions resulted in 20 hits for the period from 2020 to the present, which means 20 times the virologist Drosten is cited in court decisions. Possible duplications have not been eliminated.
